# Tributyrin and anise mixture supplementation improves growth performance, nutrient digestibility, jejunal villus height, and fecal microbiota in weaned pigs

**DOI:** 10.3389/fvets.2023.1107149

**Published:** 2023-01-27

**Authors:** De Xin Dang, Haeun Lee, Seung Jae Lee, Jun Ho Song, Seyoung Mun, Kang Yeon Lee, Kyudong Han, In Ho Kim

**Affiliations:** ^1^Department of Animal Resources Science, Dankook University, Cheonan-si, Republic of Korea; ^2^Department of Bioconvergence Engineering, Dankook University, Yongin-si, Republic of Korea; ^3^Department of Microbiology, College of Science & Technology, Dankook University, Cheonan-si, Republic of Korea; ^4^Center for Bio-Medical Engineering Core Facility, Dankook University, Cheonan-si, Republic of Korea; ^5^Semi Feed Tech Co., Ltd., Seoul, Republic of Korea

**Keywords:** tributyrin, anise, weaned pig, villus height, growth performance, nutrient digestibility

## Abstract

**Introduction:**

The objective of this study was to investigate the effects of dietary supplementation of tributyrin and anise mixture (TA) on growth performance, apparent nutrient digestibility, fecal noxious gas emission, fecal score, jejunal villus height, hematology parameters, and fecal microbiota of weaned pigs.

**Methods:**

A total of 150 21-day-old crossbred weaned pigs [(Landrace × Yorkshire) × Duroc] were used in a randomized complete block design experiment. All pigs were randomly assigned to 3 groups based on the initial body weight (6.19 ± 0.29 kg). Each group had 10 replicate pens with 5 pigs (three barrows and two gilts) per pen. The experimental period was 42 days and consisted of 3 phases (phase 1, days 1–7; phase 2, days 8–21; phase 3, days 22–42). Dietary treatments were based on a corn-soybean meal-basal diet and supplemented with 0.000, 0.075, or 0.150% TA.

**Results and discussion:**

We found that dietary supplementation of graded levels of TA linearly improved body weight, body weight gain, average daily feed intake, and feed efficiency (*P* < 0.05). TA supplementation also had positive effects on apparent dry matter, crude protein, and energy digestibility (*P* < 0.05) and jejunal villus height (*P* < 0.05). The emission of ammonia from feces decreased linearly with the dose of TA increased (*P* < 0.05). Moreover, TA supplementation was capable to regulate the fecal microbiota diversity, manifesting in a linearly increased Chao1 index and observed species and a linearly decreased Pielou's index (*P* < 0.05). The abundance of *Lactobacillus reuteri, Lactobacillus amylovorus, Clostridium butyricum* were increased, while the abundance of *Prevotella copri* was decreased, by treatment (*P* < 0.05). Therefore, we speculated that TA supplementation would improve growth performance and reduce fecal ammonia emission through improving nutrient digestibility, which was attributed to the increase of jejunal villus height and the regulation of fecal microbiota.

## Introduction

Post-weaning is a critical phase in swine husbandry. During this period, weaned pigs will encounter challenges in intestinal microflora disorder and/or gastrointestinal tract dysfunction, which will limit their digestion and absorption capacity, resulting in growth retardation and/or diarrhea (1, 2). For decades, antibiotics have been used to cope with the post-weaning challenges in swine husbandry (3). However, due to antibiotic resistance, the use of antibiotics in livestock has been considered as a threat to the safety in animal production and human health (4). For this reason, seeking suitable alternatives to antibiotics has always been the direction in animal husbandry research.

Tributyrin, also known as tributyrate glyceride, is a triglyceride containing 3 butyrate molecules, which is regarded as a precursor of butyrate (5, 6). When taken orally, it can directly release into the hindgut (7), thus exerting several biological effects such as regulating intestinal microflora (8, 9), improving intestinal health (2, 6), enhancing muscle development (10, 11), alleviating antioxidant stress (12, 13), promoting nutrients absorption (14), and ameliorating growth retardation (13, 15).

When feeding animals with a mixture of tributyrin and herb-derived extract, an increase in growth performance and a decrease in intestinal harmful bacteria were observed (16). Chen et al. (17) found that tributyrin and herb-derived extract mixture supplementation was capable to improve growth performance, maintain intestinal mucosal integrity, and regulate intestinal microbiota in weaned pigs.

Anise has long been used as an aromatic for mammals in order to induce imprinting effects (18, 19). Anise is an important traditional Chinese medicine. Bioactive compounds presented in anise, such as anethole, estragole, limonene, pinene, β-phellandrene, and α-terpineol allows it anti-inflammatory (20, 21), bacteriostatic (22, 23), antioxidant (22, 23), resist pathogenicity bacterial infection (21), and growth promotion (24, 25) characteristics. Therefore, it is reasonable to believe that the combination of tributyrin and anise has positive effects on the performance of weaned pigs.

However, no study has investigated the effects of tributyrin and anise mixture (TA) supplementation on the productive performance of pigs.

The technique of 16S rRNA high-throughput sequencing provides a reliable method for bacterial identification. The gene of 16S rRNA is a conserved sequence region that exists in all bacteria and can be targeted by broad-range polymerase chain reaction (PCR) primers (26). The 16S rRNA sequencing has been used to describe the species composition of various communities in the study of bacterial diversity (27). Therefore, we use the technique of 16S rRNA high-throughput sequencing to investigate the effects of TA supplementation on intestinal microbiota of weaned pigs. We hypothesized that feeding weaned pigs with TA containing diet had positive effects on jejunal villus height and the abundance of beneficial bacteria in intestinal microbiota, so as to improve growth performance by increasing nutrient digestibility, limiting fecal noxious gas emission, reducing fecal score.

## Materials and methods

### Experimental design

A total of 150 21-day-old crossbred weaned pigs [(Landrace × Yorkshire) × Duroc] with an average initial body weight of 6.19 ± 0.29 kg were used in a completely randomized block design experiment. The protocol (DK-1-2034) of this study was approved by the Animal Care and Use Committee of Dankook University (Cheonan, South Korea).

Based on the initial body weight, all pigs were randomly assigned to three groups. Each group had 10 replicate pens with five pigs (three barrows and two gilts) per pen. The experimental period was 42 days and divided into 3 phases (phase 1, days 1–7; phase 2, days 8–21; phase 3, days 22–42). Dietary treatments were based on a corn-soybean meal-basal diet, which were formulated to meet the nutrient requirements of the NRC (28), and supplemented with 0.000, 0.075, or 0.150% TA to form control, TRT1, and TRT2 groups. The chosen dose was determined in a preliminary study. Feed ingredients and analyzed nutrient composition of the basal diet were shown in Table 1. The commercial TA (ElanPlus^®^ TB50) used in this study was obtained from Olus Plus BV (8 Randweg, Hasselt, The Netherlands). The additive was composed of 50% tributyrin, 5% anise coated by potato starch (10% w/v), and 45% *Vehicle q.s*. (Silica).

**Table 1:** Formula and composition of experimental diet (as fed-basis).

	**Phase 1 (days 1–7)**	**Phase 2 (days 8–21)**	**Phase 3 (days 22–42)**
Ingredients, %		
Corn	37.92	48.39	58.40
Soybean meal (crude protein 47.5%)	16.44	19.40	22.27
Fermented soybean meal (crude protein 53.2%)	5.00	4.00	3.00
Spray-dried porcine plasma (crude protein 77.3%)	6.00	3.00	–
Tallow	3.32	3.08	2.83
Lactose	12.88	7.78	3.18
Sugar	3.00	3.00	3.00
Whey protein	11.00	7.00	3.00
Monocalcium phosphate	1.60	1.54	1.40
Limestone	1.12	1.06	1.06
NaCl	0.20	0.10	0.10
_DL_-Methionine (50%)	0.22	0.22	0.20
_L_-Lysine- H_2_SO_4_ (51%)	0.49	0.62	0.75
Mineral mixture^a^	0.20	0.20	0.20
Vitamin mixture^b^	0.20	0.20	0.20
Choline chloride (50%)	0.03	0.03	0.03
Zinc oxide	0.38	0.38	0.38
Total	100.00	100.00	100.00
Analyzed composition, %		
Metabolizable energy, MJ/kg	14.45	14.24	14.03
Crude protein	20.00	19.00	18.00
Calcium	0.90	0.85	0.80
Phosphorus	0.75	0.70	0.65
Lysine	1.60	1.55	1.50
Methionine	0.50	0.48	0.46
Crude fat	4.96	5.09	5.19

a Provided per kg diet: Fe, 100 mg as ferrous sulfate; Cu, 17 mg as copper sulfate; Mn, 17 mg as manganese oxide; I, 0.5 mg as potassium iodide; and Se, 0.3 mg as sodium selenite.

b Provided per kilograms of diet: vitamin A, 10,800 IU; vitamin D_3_, 4,000 IU; vitamin E, 40 IU; vitamin K_3_, 4 mg; vitamin B_1_, 6 mg; vitamin B_2_, 12 mg; vitamin B_6_, 6 mg; vitamin B_12_, 0.05 mg; biotin, 0.2 mg; folic acid, 2 mg; niacin, 50 mg; D-calcium pantothenate, 25 mg.

All pigs were housed in an environmentally controlled room. The temperature during week 1 was maintained at 30°C and then gradually reduced by 1°C every week to maintain in 24°C. The relative humidity within the room was 60%. The room was equipped with a mechanical ventilation system and the floor was slatted plastic. A nipple drinker was installed in each pen to ensure that pigs could drink freely. In addition, stainless steel self-feeders were installed on one-side of the pens to ensure that pigs had free access to feed. All pigs were allowed *ad libitum* access to feed and water.

### Feed composition analysis

On days 7, 21, and 24, representative feed samples were taken to analyze feed composition. Feed samples were dried in an oven with 70°C for 72 h, and later they were ground to pass through a 1-mm sieve and collected. Powder feed samples were analyzed for dry matter (DM; method 930.15), crude protein (CP; nitrogen × 6.25; method 968.06), calcium (method 984.01), phosphorus (method 965.17), and crude fat (method 954.02) following the procedures established by AOAC (29). The lysine and methionine contents in the diet were measured using an AA analyzer (Beckman 6300; Beckman Coulter, Inc., Fillerton, CA). The combustion heat was measured by a bomb calorimeter (Parr 6100; Parr Instrument Co., Moline, IL, USA) to determine the energy content in the feed sample.

### Experimental parameters measurement

#### Growth performance

Individual body weight of pigs was measured on days 1, 7, 21, and 42. Data of body weight was pooled on a pen basis to determine average daily gain (ADG) during days 1–7, 8–21, 22–42, and 1–42. Pen-based feed intake was measured daily to calculate the average daily feed intake (ADFI) during days 1–7, 8–21, 22–42, and 1–42. The feed efficiency (gain to feed ratio) during days 1–7, 8–21, 22–42, and 1–42 was calculated using ADG and ADFI values.

#### Apparent nutrient digestibility

During days 1–7, 14–21, and 35–42, 0.20% chromium oxide as an indigestible marker was added to the diet of each group for measuring apparent nutrient digestibility. On day 7, 21, and 42, two pigs (one barrow and one gilt) were randomly selected from each replicate pen for fecal taking (about 250 g) by the rectal massage method. Fecal samples were dried in an oven with 70°C for 72 h. After that, samples were ground into powder, which can pass through a 1-mm sieve, and be collected in duplicate. Fecal samples were analyzed for DM (method 930.15) and CP (nitrogen × 6.25; method 968.06) following the procedures established by AOAC (29). The combustion heat was measured by a bomb calorimeter (Parr 6100; Parr Instrument Co., Moline, IL, USA) to determine the energy in feces. The chromium levels were analyzed *via* UV absorption spectrophotometry (UV-1201, Shimadzu, Kyoto, Japan). The apparent total tract digestibility was calculated according to the equation provided by Liu and Kim (30).

#### Jejunal villus height

Two pigs per pen (one barrow and one gilt) were selected randomly and euthanized with an intravenous injection of 3 mg/kg body weight of chlorpromazine hydrochloride injection on day 42. The entire intestine of euthanized pigs was then removed and dissected free of mesenteric attachments and placed on a smooth and cold surface. The jejunum was separated. The isolated intestinal segments were immediately opened lengthwise following the mesentery line and flushed with ice-cold saline. Approximately 2 cm segments of the jejunum at consistent locations were collected immediately, fixed in 10% formalin, then subsequently embedded, sectioned and stained with hematoxylin and eosin by routine methods. Villus height of the jejunum was measured in ~10 microscopic fields using an image analysis system by a blinded investigator.

#### Fecal score

Fecal scores of pigs were recorded daily using the scoring system proposed by Hashem and Shehata (31) in the first week post-weaning.

#### Hematology parameters

On the last day of the experiment, before euthanizing the pigs used above, they were bled for collecting blood samples *via* anterior vena cava puncture into non-heparinized vacuum tubes (Becton Dickinson Vacutainer Systems, Franklin Lakes, NJ, USA). The blood samples were collected during 11:00 to 12:00 h in order to exclude the circadiurnal fluctuations in hormone concentrations. Pigs did not receive any feed before sampling. Blood samples were centrifuged (3,000 × *g*) for 15 min at 4°C to obtain serum samples and then stored at −20°C until analysis. The concentrations of total protein and albumin were assayed using colorimetric methods. Additionally, globulin levels were evaluated through the difference between total protein and albumin. Serum total cholesterol, triglyceride, and high-density lipoprotein cholesterol (HDL-C) concentrations were determined enzymatically using reagent kits (Wako Pure Chemical Industries Ltd., Tokyo, Japan).

#### Fecal noxious gas emission

On day 7, 21, and 42, the method of rectal massage was used to take fecal samples (about 300 g) from two randomly selected pigs (one barrow and one gilt) in each pen for measuring fecal ammonia (NH_3_), hydrogen sulfide (H_2_S), acetic acid, carbon dioxide (CO_2_), and total mercaptans (R-SH) emission by the method provided by Dang et al. (32). The fecal samples from the same pen were pooled, mixed, and transported to lab for further analysis of fecal noxious gas emission.

#### Metagenomic DNA extraction & 16S rRNA gene V3–V4 amplicon sequencing

Fresh stool samples were taken from 15 pigs (five pigs/group) after feeding phytogenic TA additives for 6 weeks. The specimens were kept in liquid nitrogen until they arrived at the laboratory. Metagenomic DNA (mDNA) from 15 fecal samples was extracted using QIAamp Power Fecal Kit (Qiagen, Germany). The mDNA extraction experimental steps were performed according to the manufacturer's instructions with the inclusion of a homogenization step in which 100 mg fecal samples were pooled in 1.4 ml lysis buffer. When the samples were thoroughly homogenized, tissue lysis stages were implemented for 6 min at 30 Hz, followed by the ending process at 95°C for 5 min. The extracted mDNA was eluted in 100 μl with buffer provided in the kit. Thereafter, the quality check of all mDNA samples was conducted using NanoDrop One (ThermoFisher Scientific, USA), and all samples were stored at 4°C until the next process. All PCR steps were performed with 2X KAPA HiFi Hot Start Ready Mix (Roche, Germany). A primer pair suitable for the 16S V3–V4 amplification was used, and the sequences were as follows: 341F forward primer is 5′-TCGTCGGCAGCGTCAGATGTGTATAAGAGACAG-3′. 806R reverse primer is 5′-GTCTCGTGGG CTCGGAGATGTG TATAAGAGACAG-3′. After PCR amplification, all amplicons were purified with the AMPure XP beads (Beckman Coulter, USA). The second PCR amplification was then conducted at lower cycles to add the Illumina adapter and multiplexing indices included in Nextera XD Index (Illumina, USA). All second PCR products were purified with AMPure XP beads once again. The final amplicon products were pooled with normalized concentration, and the library size was checked using the TapeStation system (Agilent, CA, USA). Finally, high-throughput amplicon sequencing was carried out using the Illumina Miseq™ paired-end (2 × 300) platform (Illumina, USA).

#### Microbial 16S V3-V4 sequencing data pre-processing

Sequencing reads created from microbial 16S V3–V4 regions were demultiplexed by the split_libraries_fastq.py function of QIIME 2, a next-generation microbiome bioinformatics platform (33). The sequences were then trimmed using the Divisive Amplicon Denoising Algorithm 2 (DADA2) plugin which detects and corrects amplicon errors. Also, sequence quality was controlled with DADA2 by filtering out PhiX chimeric sequences. Sequences including ambiguous base calls and <100 bp were trimmed to minimize the random errors. After the denoising step, the data was specified with pre-trained Naïve Bayes classifier artifact using the machine learning Python library scikit-learn in the QIIME2 pipeline. The classifier artifact was trained on SILVA database v138 which is trimmed to include only V3–V4 regions, pre-clustered with 99% sequence identity, and classified bacterial taxonomy using a 70% confidence threshold (default).

#### Alpha and beta diversity analyses

For all samples, alpha and beta diversity analyses were performed with the “diversity” QIIME2 algorithm to measure changes or differences in microorganisms. The alpha-diversity was estimated by observed ASVs, Chao1, Shannon's abundance, and Pielou's evenness indices, indicating microbial richness and evenness measures within a single sample. The Kruskal–Wallis non-parametric test was then calculated to identify statistical differences among the three groups. The beta-diversity measures were estimated using principal coordinate analysis (PCoA) of both Unweighted UniFrac and Bray-Curtis abundance dissimilarity metrics with the non-parametric permutational multivariate analysis of variance (PERMANOVA) test which is used to compare the differences between the groups. All non-parametric statistical analyses were conducted by GraphPad PRISM v8 (GraphPad Software. Inc., CA, USA), and the *P*-value < 0.05 was considered statistically significant. All visualization data was created using R bioinformatics packages.

#### Relative abundance analysis

A relative abundance analysis was carried out to determine the relative frequency of bacteria from phylum to species level of each group. The Kruskal–Wallis non-parametric test was also calculated to confirm statistical differences among three groups. Furthermore, we visualized the statistically significant species of the three groups with the Venn diagram (*P* < 0.05). The heatmap visualization was performed on 15 species among the shared species shown in the Venn diagram except for the unclassification and uncultured bacteria using R studio version 4.1.2. Finally, we compared the relative frequency of the representative four species to analyze the linear effects of the phytogenic additive on the weaning pigs.

### Statistical analysis

Before the analysis, all the percentage data were transformed by arcsine transformations. Data were then subjected to statistical analysis in a randomized complete block design using the General Linear Models procedures (SAS Institute, Cary, NC, USA). The normality of data was examined by the Shapiro–Wilk test and QQ plots. The replicate pen was used as the experimental unit. Orthogonal polynomials were used to assess the linear and quadratic effects of storage duration. Differences among groups were evaluated by the one-way ANOVA for multiple comparisons. Variability in the data was expressed as the pooled standard error of means (SEM). *P* < 0.05 was considered statistically significant.

## Results

### Growth performance and fecal score

In comparison to control group, feeding weaned pigs with 0.15% TA containing diet led to higher body weight on day 42 (*P* = 0.038), ADG during days 8–21 (*P* = 0.012), 22–42 (*P* = 0.009), and 1–42 (*P* = 0.006), ADFI during days 22–42 (*P* = 0.040) and 1–42 (*P* = 0.028), and feed efficiency during days 1–42 (*P* = 0.034). Above growth performance parameters were increased linearly with the dose of TA increased. Moreover, no significant difference in fecal score has been observed among groups (Table 2).

**Table 2:** Effects of tributyrin and anise mixture (TA) supplementation on growth performance and fecal score of weaned pigs^1^.

**Items**	**TA, %**			**SEM**	***P*** **-value**		
	**0.000**	**0.075**	**0.150**		**ANOVA**	**Linear**	**Quadratic**
**Body weight, kg**							
Day 1	6.18	6.19	6.19	0.294	1.000	0.985	0.998
Day 7	8.01	8.08	8.06	0.293	0.979	0.911	0.896
Day 21	13.96	14.23	14.30	0.299	0.611	0.426	0.783
Day 42	24.53^b^	25.24^ab^	25.58^a^	0.338	0.041	0.038	0.660
**ADG, g**							
Days 1–7	261.23	271.03	266.80	4.302	0.164	0.368	0.194
Days 8–21	425.01^b^	439.51^ab^	446.09^a^	5.508	0.008	0.012	0.562
Days 22–42	503.37^b^	520.39^ab^	533.23^a^	7.479	0.007	0.009	0.821
Days 1–42	436.89^b^	453.88^ab^	461.26^a^	5.828	0.004	0.006	0.507
**ADFI, g**							
Days 1–7	292.86	301.43	297.14	4.511	0.280	0.508	0.255
Days 8–21	526.57	541.29	545.14	7.769	0.118	0.052	0.484
Days 22–42	706.48^b^	719.52^ab^	734.00^a^	11.013	0.097	0.040	0.950
Days 1–42	577.57^b^	590.43^ab^	598.24^a^	7.637	0.081	0.028	0.741
**Feed efficiency** ^2^							
Days 1–7	0.89	0.90	0.90	0.007	0.692	0.588	0.646
Days 8–21	0.81	0.81	0.82	0.007	0.368	0.255	0.930
Days 22–42	0.71	0.72	0.73	0.007	0.237	0.186	0.624
Days 1–42	0.76^b^	0.77^ab^	0.77^a^	0.006	0.070	0.034	0.349
Fecal score	1.17	1.15	1.14	0.058	0.924	0.694	0.968

ADG, average daily gain; ADFI, average daily feed intake; SEM, standard error of the mean.

^1^Values represent the means of 10 pens with 5 pigs per replicate pen (n = 10) per treatment.

^2^Gain to feed ratio.

^a, b^Means in the same row with different superscript differ significantly (P < 0.05).

### Apparent nutrient digestibility and jejunal villus height

**Figure 1:**
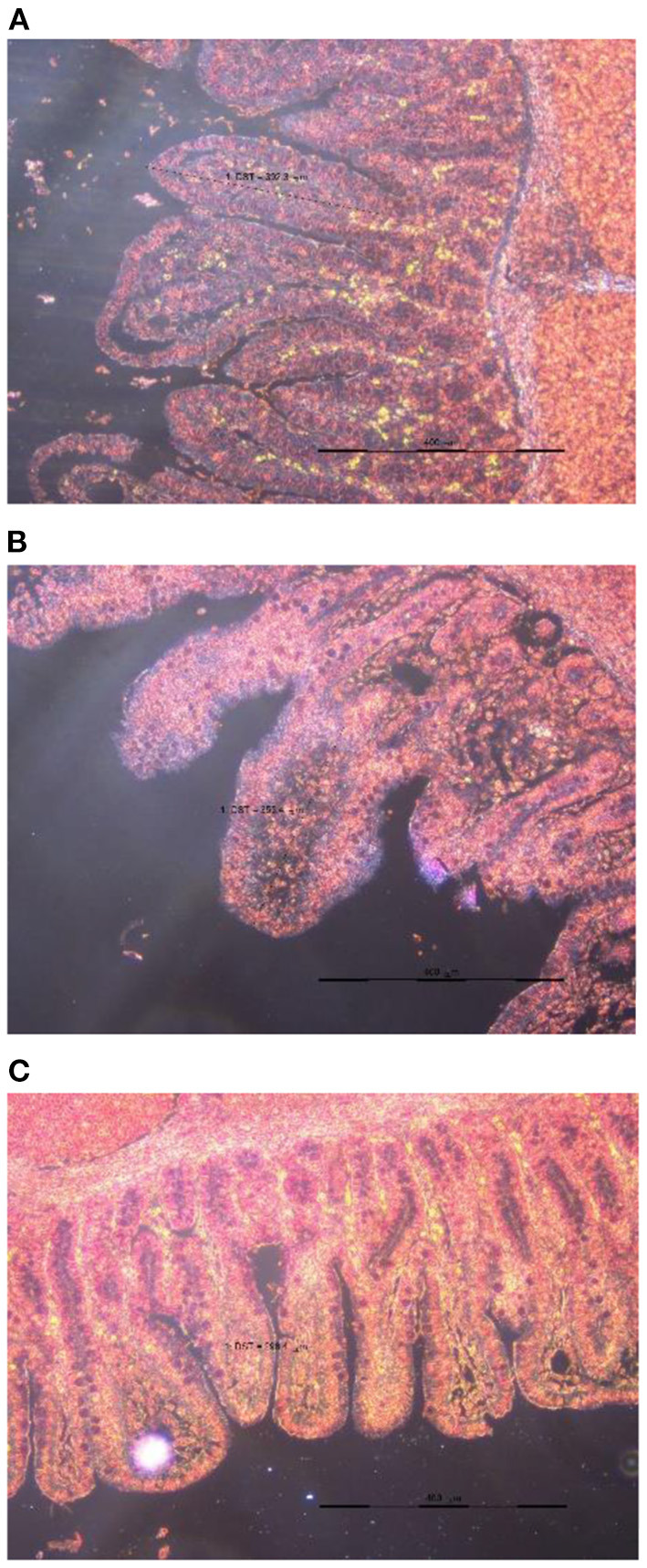
Slice of the jejunum in weaned piglets fed the diet supplemented 0.000 **(A)**, 0.075 **(B)**, or 0.150 **(C)** % tributyrin and anise mixture.

Apparent DM digestibility on day 7 (*P* = 0.008), day 21 (*P* = 0.043), and 42 (*P* = 0.029), apparent CP digestibility on day 42 (*P* = 0.033), and apparent energy digestibility on day 7 (*P* = 0.002), day 21 (*P* = 0.028), and 42 (*P* = 0.010) were the highest in the group of TRT2 in comparison to other groups and they were increased linearly with the dose of TA increased in the diet. Additionally, feeding weaned pigs with graded levels of TA containing diet increased jejunal villus height (Figure 1) in a dose-dependent manner (linearly; *P* = 0.043), of which the highest value was presented in TRT2 group (Table 3).

**Table 3:** Effects of tributyrin and anise mixture (TA) supplementation on apparent nutrient digestibility and jejunal villus height of weaned pigs^1^.

**Items**	**TA, %**			**SEM**	***P*** **-value**		
	**0.000**	**0.075**	**0.150**		**ANOVA**	**Linear**	**Quadratic**
**DM, %**							
Day 7	79.91^b^	81.01^ab^	81.75^a^	0.639	0.013	0.008	0.749
Day 21	82.15^b^	82.37^b^	83.73^a^	0.674	0.087	0.043	0.398
Day 42	84.55^b^	86.30^ab^	86.51^a^	0.848	0.033	0.029	0.309
**CP, %**							
Day 7	78.09	78.67	80.60	1.331	0.116	0.071	0.567
Day 21	81.31	81.49	82.51	1.251	0.119	0.079	0.466
Day 42	82.02^b^	84.16^ab^	85.22^a^	1.423	0.059	0.033	0.665
**Energy, %**							
Day 7	79.12^b^	79.77^b^	81.23^a^	0.626	0.003	0.002	0.465
Day 21	81.38^b^	82.28^ab^	83.56^a^	0.651	0.004	0.028	0.813
Day 42	83.44^b^	85.19^ab^	85.65^a^	0.794	0.012	0.010	0.358
Villus height, μm	301.97^b^	344.23^ab^	353.21^a^	18.556	0.095	0.043	0.438

DM, dry matter; CP, crude protein; SEM, standard error of the mean.

^1^Values represent the means of 10 pens with 2 pigs per replicate pen (n = 10) per treatment.

^a, b^Means in the same row with different superscript differ significantly (P < 0.05).

### Hematology parameters

The concentrations of serum total protein, albumin, globulin, cholesterol, triglyceride, and HDL-C did not differ among groups (Table 4).

**Table 4:** Effects of tributyrin and anise mixture (TA) supplementation on hematology parameters of weaned pigs^a^.

**Items**	**TA, %**			**SEM**	***P*** **-value**		
	**0.000**	**0.075**	**0.150**		**ANOVA**	**Linear**	**Quadratic**
Total protein, g/dl	5.30	5.48	5.18	0.313	0.796	0.791	0.543
Albumin, g/dl	2.92	2.68	2.78	0.097	0.253	0.327	0.178
Globulin, g/dl	2.38	2.80	2.40	0.305	0.563	0.964	0.294
Cholesterol, mg/ml	83.20	87.40	80.40	4.073	0.494	0.636	0.284
Triglyceride, mg/ml	53.40	53.40	55.60	6.112	0.958	0.803	0.886
HDL-C, mg/ml	30.20	30.60	26.80	2.101	0.401	0.275	0.430

HDL-C, high-density lipoprotein cholesterol; SEM, standard error of the mean.

^a^Values represent the means of 10 pens with two pigs per replicate pen (n = 10) per treatment.

### Fecal noxious gas emission

Weaned pigs fed the diet supplemented with TA linearly decreased fecal NH_3_ emission on day 42 (*P* = 0.040), while did not affect the emissions of H_2_S, R-SH, acetic acid, and CO_2_. Moreover, the emission of NH_3_ in control group was lower than that in TRT2 group (Table 5).

**Table 5:** Effects of tributyrin and anise mixture (TA) supplementation on fecal noxious gas emission of weaned pigs^1^.

**Items**	**TA, %**			**SEM**	***P*** **-value**		
	**0.000**	**0.075**	**0.150**		**ANOVA**	**Linear**	**Quadratic**
**NH**_3_ **, ppm**							
Day 7	1.13	1.38	1.13	0.267	0.516	1.000	0.256
Day 21	1.38	1.25	1.13	0.317	0.701	0.404	1.000
Day 42	1.75^a^	1.38^ab^	1.25^b^	0.247	0.098	0.040	0.534
**H**_2_ **S, ppm**							
Day 7	1.00	1.05	0.98	0.187	0.908	0.887	0.681
Day 21	1.05	1.15	1.05	0.206	0.834	1.000	0.552
Day 42	1.30	1.35	1.38	0.225	0.935	0.722	0.945
**R-SH, ppm**							
Day 7	2.38	2.25	1.88	0.377	0.348	0.167	0.684
Day 21	1.75	1.88	1.63	0.377	0.776	0.724	0.542
Day 42	2.13	1.75	1.88	0.429	0.636	0.536	0.475
**Acetic acid, ppm**							
Day 7	6.63	7.25	6.75	0.545	0.438	0.807	0.212
Day 21	6.88	6.75	6.50	0.498	0.714	0.425	0.877
Day 42	6.75	7.13	7.13	0.429	0.561	0.356	0.591
**CO**_2_ **, ppm**							
Day 7	9,775.00	9,600.00	9,275.00	262.202	0.137	0.052	0.725
Day 21	10,050.00	10,100.00	10,050.00	435.890	0.990	1.000	0.888
Day 42	10,225.00	10,175.00	10,575.00	257.256	0.211	0.157	0.288

NH_3_, ammonia; H_2_S, hydrogen sulfide; R-SH, total mercaptans; CO_2_, carbon dioxide; SEM, standard error of the mean.

^1^Values represent the means of 10 pens with 2 pigs per replicate pen (n = 10) per treatment.

^a, b^Means in the same row with different superscript differ significantly (P < 0.05).

### Alpha and beta diversity in fecal microbiota

The average number of total raw reads for 16S metagenome sequencing was 189,215 and the average number of filtered reads, denoised reads, merged reads, and non-chimeric reads were 39,551, 35,450, 23,394, and 14,794, respectively. We confirmed that non-chimeric read data was sufficient enough to analyze the microbiome and the number of ASVs taxonomy classified by SILVA v138 reference database with a >70% confidence threshold was 1,045.

We performed the alpha-diversity analysis to analyze in abundance and evenness differences of each group. The alpha-diversity was measured using Chao1 index, Observed species, Shannon index, and Pielou's index (Table 6). As a result of diversity estimation, observed Chao1 index (*P* = 0.042) and observed species (*P* = 0.044) measures linearly increased when feeding weaned pigs with graded levels of TA containing diet. In contrast, Pielou's evenness estimated scores linearly decreased with the dose of TA increased, showing a significant statistical value (*P* = 0.020). Additionally, the highest value of Chao1 index and Observed species were presented in TRT2 group, whereas the lowest value of Pielou's index was presented in TRT1 and TRT2 groups, in comparison to other groups.

**Table 6:** Effects of tributyrin and anise mixture (TA) supplementation on diversity and abundance indexes in fecal microbiota of weaned pigs^1^.

**Items**	**TA %**			**SEM**	***P*** **-value**		
	**0.000**	**0.075**	**0.150**		**ANOVA**	**Linear**	**Quadratic**
Chao1 index	173.25^b^	232.38^ab^	254.09^a^	25.102	0.036	0.042	0.554
Observed species	171.60^b^	228.80^ab^	247.40^a^	23.828	0.037	0.044	0.521
Shannon index	6.60	6.68	6.80	0.149	0.519	0.360	0.939
Pielou's index	0.89^a^	0.86^b^	0.86^b^	0.009	0.004	0.020	0.068

SEM, standard error of the mean.

^1^Values represent the means of 5 pigs per treatment (n = 5).

^a, b^Means in the same row with different superscript differ significantly (P < 0.05).

**Figure 2:**
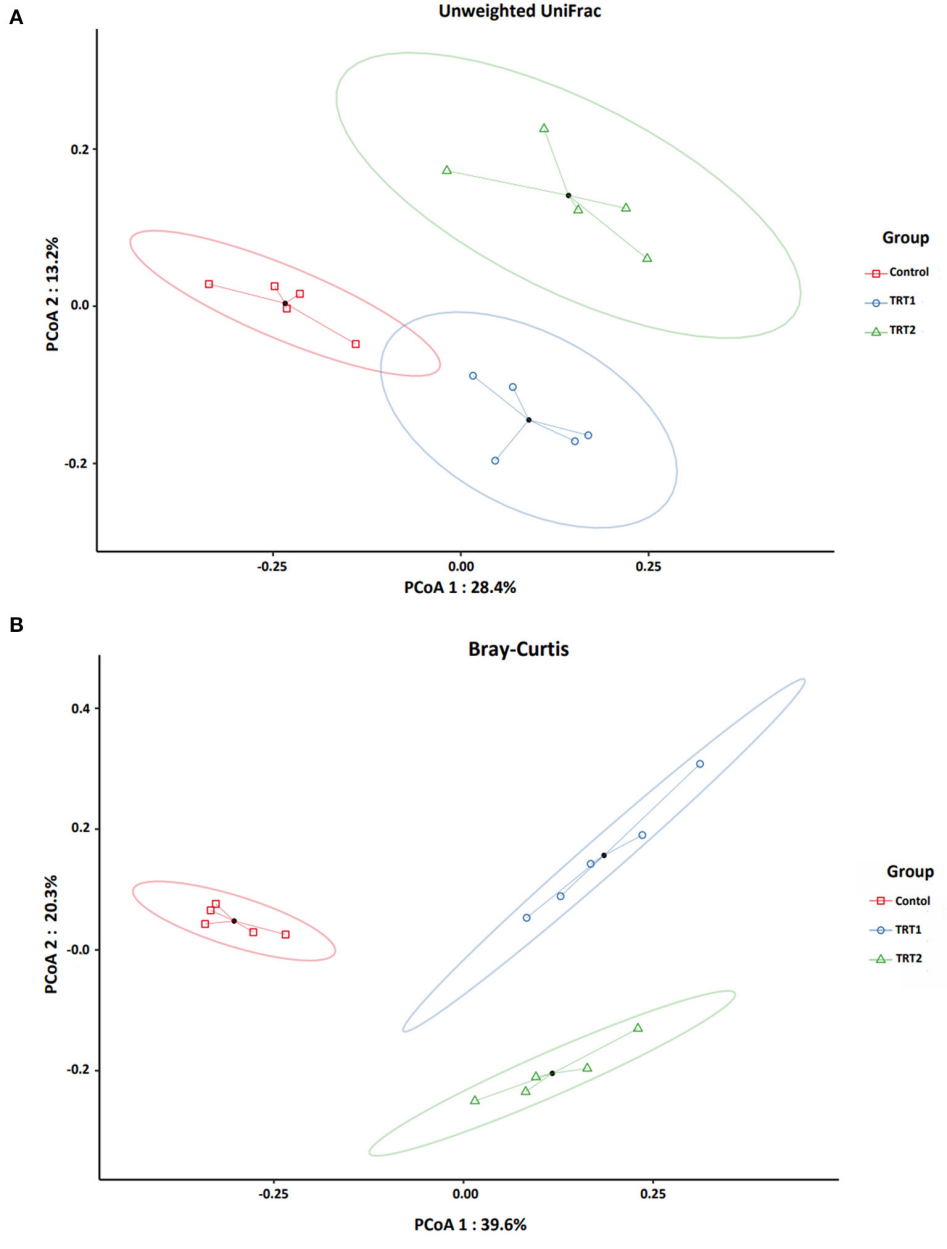
Beta-diversity analysis of the three groups between each weaning pigs (*n* = 5). Microbial beta-diversity analysis of the three groups is measured by phylogenetic qualitative Unweighted UniFrac **(A)** and Bray-Curtis **(B)** abundance dissimilarity distance matrix for all 15 samples. The colored circular clusters denote the control (red), TRT1 (blue), and TRT2 (green). Control was defined as the weaned pigs fed the basal diet. TRT1 was defined as the weaned pigs fed the diet supplemented with 0.075% tributyrin and anise mixture. TRT2 was defined as the weaned pigs fed the diet supplemented with 0.150% tributyrin and anise mixture.

We analyzed beta-diversity to affirm the change of species in each group by coordinates analysis (PCoA) using phylogenetic qualitative Unweighted UniFrac (A) and Bray-Curtis (B) abundance dissimilarity distance (Figure 2). We found that both indices showed statistical importance (*P* = 0.001) based on the PERMANOVA test. In addition, pseudo-F, which indicates the ratio of the between-cluster variations to the within-cluster variation, showed higher scores in Bray-Curtis than Unweighted UniFrac (Table 7).

**Table 7:** Statistical significance of beta-diversity using PERMANOVA-test.

**Metric name**	**pseudo-*F***	***R* ^2^**	***P*-value**
Unweighted UniFrac	3.70845919	0.381982261	0.001
Bray-Curtis	7.641977174	0.560181056	0.001

### Relative abundance in fecal microbiota

**Figure 3:**
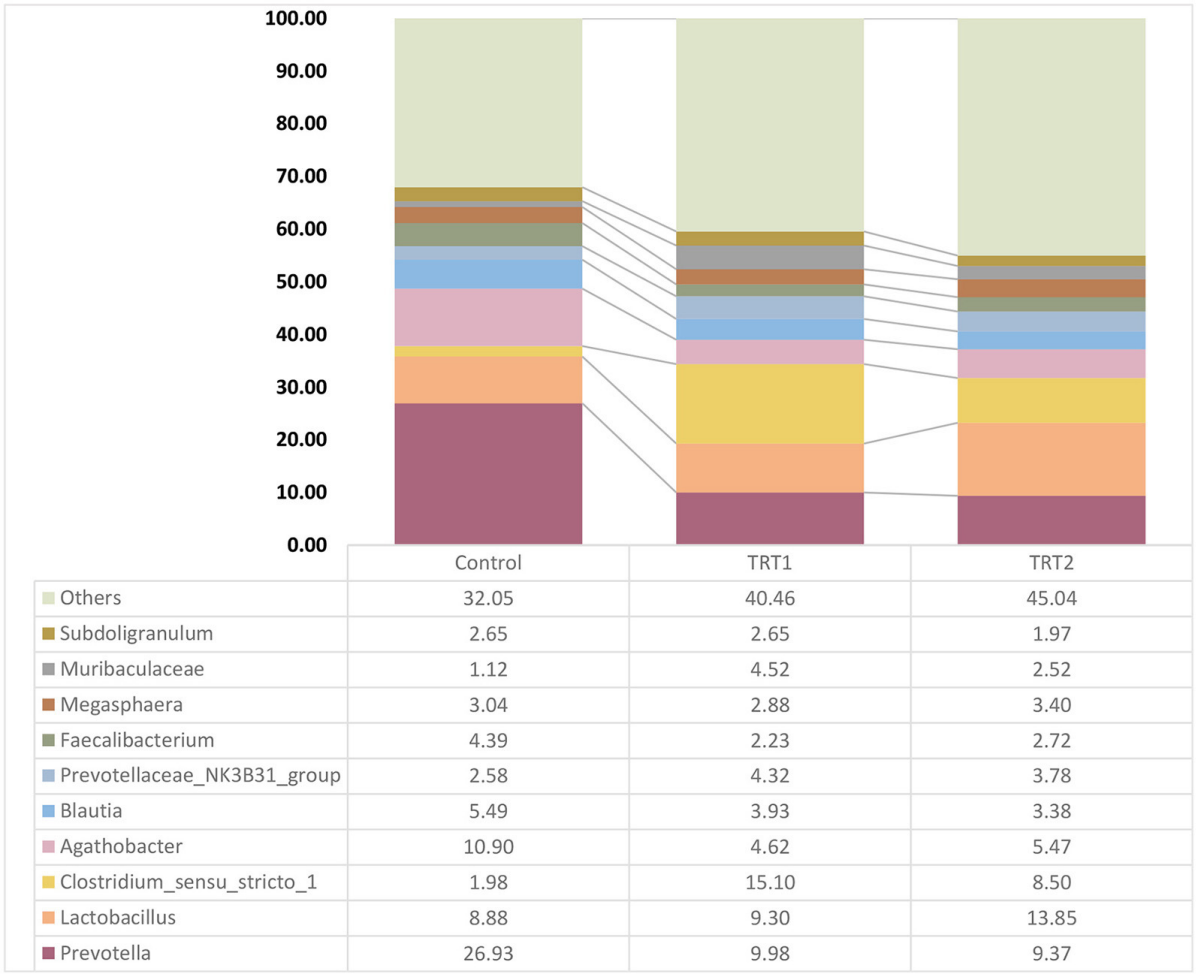
Relative bacterial abundance of the groups at the genus level (*n* = 5). The relative abundance box plots indicate the composition of the three groups at the genus levels which are classified based on the 16S V3–V4 metagenome profiling. Each graph bar is ordered from the highest proportion. Control was defined as the weaned pigs fed the basal diet. TRT1 was defined as the weaned pigs fed the diet supplemented with 0.075% tributyrin and anise mixture. TRT2 was defined as the weaned pigs fed the diet supplemented with 0.150% tributyrin and anise mixture.

We confirmed a relative bacterial frequency at the genus level based on the taxonomy classification results using SILVA v138 16S rRNA gene database (Figure 3). The 10 most abundant bacteria among groups were mainly involved in *Subdoligranulum, Muribaculaceae, Megasphaera, Faecalibacterium, Prevotellaceae_NK3B31_group, Blautia, Agathobacter, Clostridium_sensu_stricto_1, Lactobacillus*, and *Prevotella*.

**Figure 4:**
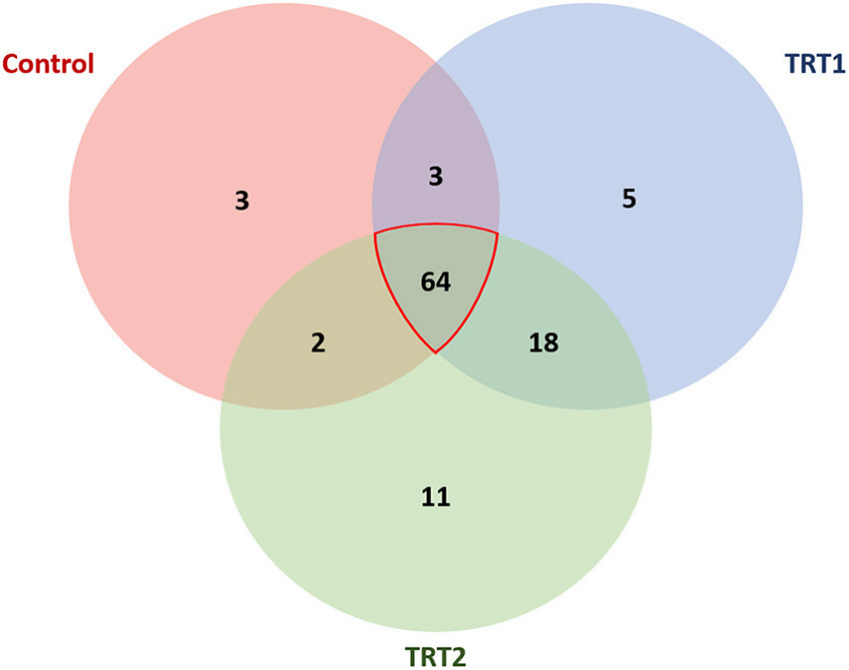
Venn diagram visualization at the species level. The Venn diagram represents the species with statistical significance in the three groups. Among the 106 species, we select 64 species shared by all three groups to analyze the linear effect of the additive. Control was defined as the weaned pigs fed the basal diet. TRT1 was defined as the weaned pigs fed the diet supplemented with 0.075% tributyrin and anise mixture. TRT2 was defined as the weaned pigs fed the diet supplemented with 0.150% tributyrin and anise mixture.

**Figure 5:**
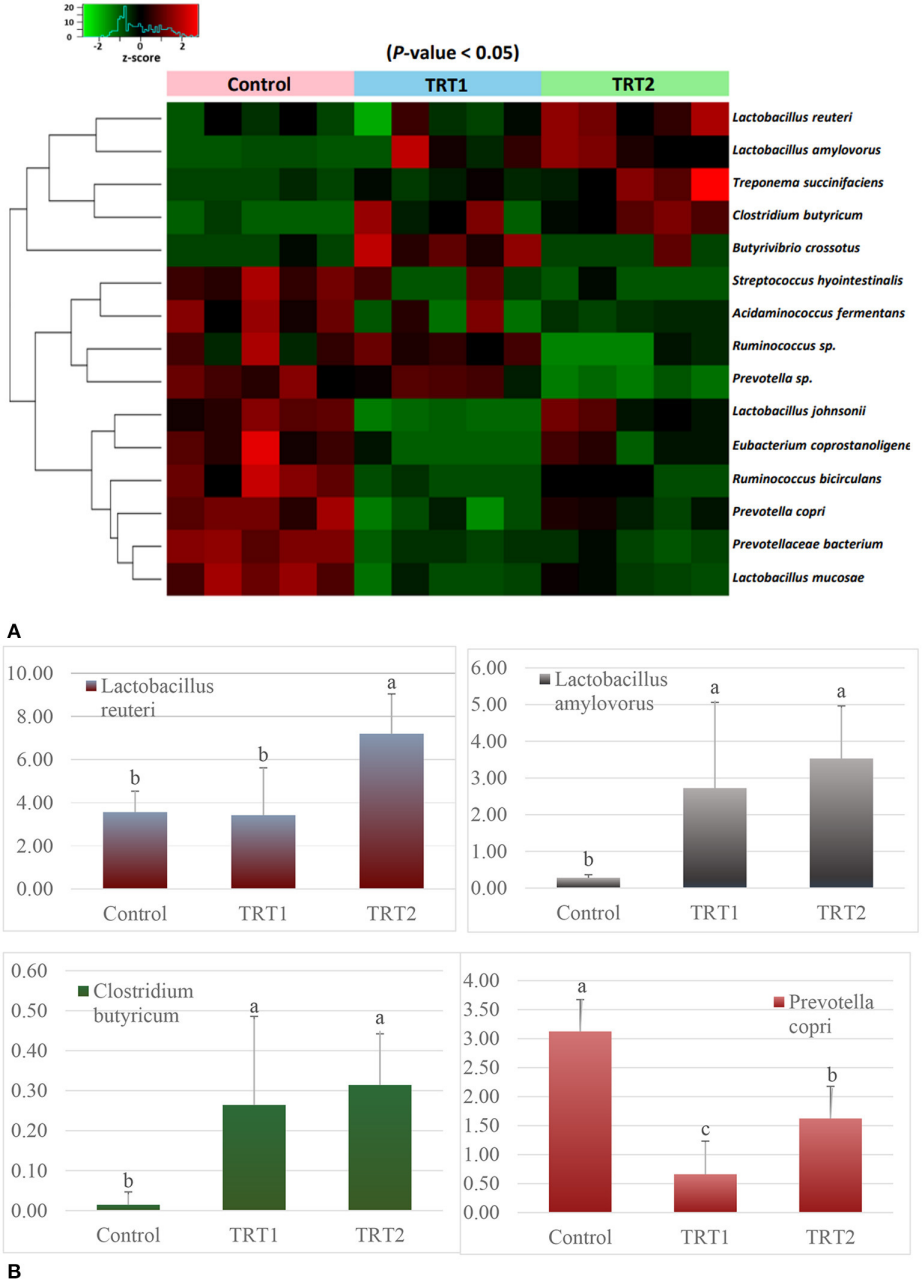
Analysis of bacterial relative abundance at the species level (*n* = 5). **(A)** Heatmap plot shows the changes in the relative abundance of the shared 15 species of each weaning pig except for ambiguous species. The relative frequency of the shared bacteria is converted to *z*-score value. **(B)** A bar chart presents the four species' relative frequency. Control was defined as the weaned pigs fed the basal diet. TRT1 was defined as the weaned pigs fed the diet supplemented with 0.075% tributyrin and anise mixture. TRT2 was defined as the weaned pigs fed the diet supplemented with 0.150% tributyrin and anise mixture. ^a − c^Means in the same figure with different superscript differ significantly (*P* < 0.05).

In order to accurately determine the effects of the TA diets on the gut microbiome, we first selected the species showing statistical significance, and 106 species were identified in the three groups (Supplementary Table 1). Then, we identified the species of each group using the Venn diagram and classified three species, five species, and 11 unique species in the control, TRT1, and TRT2 groups, respectively. There were also three species shared between the control and TRT1 groups, common two species between the control and the TRT2, and five species between the TRT1 and TRT2 groups. In line with the purpose of the study, we focused on 64 common species in the three groups (Figure 4). Finally, we compared the relative abundance of the 15 species, which was converted into a *z*-score using a heatmap, excluding ambiguous species (e.g., unclassification or uncultured_bacterium; Figure 5A). Among the 15 species, we picked representative four species to investigate the effects of the dietary additive. The abundance of *Lactobacillus reuteri* in TRT2 group was higher than that in control and TRT1 groups (*P* < 0.05). The abundance of *Lactobacillus amylovorus* and *Clostridium butyricum* in TRT1 and TRT2 groups were higher than that in control group (*P* < 0.05). Moreover, the abundance of *Prevotella copri* was significantly reduced by treatment, of which higher value was observed in TRT2 group in compared to that in TRT1 group (Figure 5B).

## Discussion

In the present study, feeding weaned pigs with graded levels of TA containing diet had positive effects on growth performance. Body weight and ADG as important economic parameters are closely related to ADFI and feed efficiency. A high ADFI ensures a good nutrition supply. A high feed efficiency allows good absorption of nutrients from the feed. It is reported that tributyrin supplementation was capable to regulate appetite (8, 13, 34). Gu et al. (8) reported that feeding lipopolysaccharide-challenged weaned pigs with 0.6 g/kg tributyrin containing diet prevented growth retardation by stimulating appetite. Wang et al. (13) noted that dietary supplementation of 0.75 g/kg tributyrin increased ADFI and ADG in diquat-challenged weaned pigs. However, the mechanism of tributyrin promoting feed intake is still unclear, which is probably related to butyrate systemic circulation mediated by tributyrin (7, 35, 36). In addition, dietary supplementation of aromatic substances has been reported to increase the appetite, thus promoting voluntary feed intake and further improving growth performance (25, 37). The flavor of trans-anethole is described as sweet (38), and its chemosensory features could improve palatability and feed preferences of weaned pigs by exerting pleasant sensations (37). Charal (23) reported that feeding weaned pigs with 50 mg/kg anise oil containing diet had positive effects on feed intake and growth performance. Dang et al. (19) noted that the ADG of weaned pigs increased by anise-containing mixture supplementation, which was attributed to the promotion of feed intake. Therefore, as the components of TA, the tributyrin and anise were capable to regulate appetite, which partially contributed to the improvement of growth performance. Additionally, Hou et al. (14) reported that feeding 5 g/kg tributyrin containing diet to weaned pigs was capable to increase feed efficiency and then improve their body weight. Sotira et al. (39) noted that weaned pigs fed the diet supplemented with 2 g/kg tributyrin increased feed efficiency, thus improving ADG. In addition, the supplementation of biochemical substances from anise also has been reported to improve the feed efficiency of weaned pigs (21, 23). Charal (23) reported that the supplementation of anise oil increased the growth of weaned pigs in nursery stages, and had a positive effect on feed efficiency. Yi et al. (21) noted that supplementing 300 mg/kg anethole to Enterotoxigenic *Escherichia coli*-infected weaned pigs increased feed efficiency. The improvement of digestibility, utilization rate, and retention rate of energy and nutrients partly explain the improvement of feed efficiency in pigs (40, 41). In this study, weaned pigs fed the diet supplemented with TA had positive effects on apparent nutrient digestibility. Similarly, some studies have reported that the supplementation of tributyrin was beneficial to improve nutrient digestibility in weaned pigs (42–44). However, no study has investigated the effects of anise supplementation on nutrient digestibility in weaned pigs. Therefore, we considered that dietary supplementation of TA had positive effects on nutrient digestibility, feed efficiency, and ADFI, and therefore contributed to the improvement in body weight and ADG.

It is reported that manipulating intestinal microbiota communities is a strategy to improve nutrient digestibility and/or feed efficiency (45, 46). Our diversity (alpha and beta) results demonstrated that the TA treatment explicitly influenced the composition of the weaning pigs' gut microbiome, especially in the aspects of evenness and abundance. The classification results at the genus level showed that the microbiome transition was related to additive treatments to some extent. However, species-level classification was further necessary because the genera have functions in both beneficial and pathogenic ways depending on the specific species. Therefore, we first selected 64 species shared by the three groups and selected 15 species showing statistical significance except for unclassified and uncultured bacteria. Finally, four representative species were analyzed to measure the effects of the additive. We found that the abundance of *L. reuteri, L. amylovorus*, and *C. butyricum* were positively, whereas *P. copri* was negatively, affected by treatment. A substantial number of studies have shown that *L. reuteri* and *L. amylovorus* in the intestine of pigs had positive effects on growth performance and intestinal health (e.g., gut integrity, nutrient digestion) (47–49). In particular, *L. reuteri* is crucial commensal bacteria in pigs that produce exopolysaccharides to increase intestinal adhesion and inhibit the proliferation of *E. coli*, thereby promoting growth performance (50, 51). Further, reutericyclin, produced by *L. reuteri*, is capable to prevent *Clostridium difficile* infection (52). Shen et al. (49) also reported that *L. amylovorus* showed the characteristics of probiotics in livestock production. Based on these previous studies, we speculated that the additive had a positive effect on weaned pigs by increasing *Lactobacillus* species. In addition to the two species, we also found a more than 20-fold increase in *C. butyricum* and an approximately half decrease in *P. copri* in the treatment groups. Studies have examined the positive effects of *C. butyricum* on intestinal morphology, intestinal microflora balance, and growth performance (53, 54). Moreover, *Clostridium butyiricum*, a butyric acid producer, is capable to provide energy to intestinal epithelium (55) and beneficial to improve nutrient digestibility (56). Regarding *P. copri*, it is capable to induce dysbiosis, reduce short-chain fatty acids, and increase the risk of colitis (57–61). Additionally, research indicated that the *P. copri* is a potential pathogen that causes a series of metabolic diseases (62). Therefore, the increase of beneficial bacteria such as *L. reuteri, L. amylovorus*, and *C. butyricum* as well as the decrease of harmful bacteria such as *P. copri* caused by TA supplementation was beneficial to the improvement of nutrient digestibility and/or feed efficiency, and therefore contributed to the improvement of growth performance.

On the other hand, the physiology of the intestinal tract is the cornerstone for ensuring high nutrient digestibility and/or feed efficiency (63, 64). The villus height condition is related to the ability of nutrient absorption (42, 65). The supplementation of tributyrin to the diet has been reported to increase the villus height in the intestine of weaned pigs (2, 8, 13, 66, 67). Tributyrin is a strong mitosis promoter and a differentiation agent in the growth of villi in gastrointestinal tract (7, 68), which is considered to be the mechanism by which the height of intestinal villus increases (65). In addition, feeding *E. coli* K88-challenged weaned pigs with 300 mg/kg anethole containing diet has been reported to increase the villus height in the duodenum, thus increasing feed efficiency (21). Taken together, these data indicated that both tributyrin and anise bioactive components have the ability to enhance the intestinal absorption of nutrients by increasing villus height.

Feeding manipulation to alleviate post-weaning diarrhea is also a strategy to improve growth performance (69). It has been reported that dietary supplementation of tributyrin decreased fecal score during the post-weaning (67). However, Zhang et al. (70) noted that feeding weaned pigs with 1 g/kg tributyrin containing diet did not affect the diarrhea rate. In this study, pigs were healthy and showed no signs of diarrhea. In addition, results of 16S rRNA analysis indicated that pig feces were rich in probiotics, such as *Prevotella* (71), *Lactobacillus* (72), *Agathobacter* (73), *Blautia* (71), Prevotellaceae_NK3B31_group (74), *Faecalibacterium* (75), *Megasphaera*

(76), and *Muribaculaceae* (77). Indeed, the efficacy of tributyrin varies according to the health status of animals (15). In addition, Hamer et al. (78) reported the dual effect of butyrate on intestinal epithelial permeability, that is, low dose of butyrate enhanced tight junction and decreased intestinal permeability *in vitro* and *in vivo* in pigs, while high concentration of butyrate increased permeability both in some intestinal cell lines and in murine models. Therefore, we considered that the effect of tributyrin on fecal score was variable. Its supplementation may not be useful for pigs with no diarrhea symptoms. Studies that evaluated the effects of anise supplementation on fecal score in weaned pigs are still limited. In this study, we considered that the supplementation of TA did not affect the fecal score in weaned pigs, which was probably due to the lack of diarrhea symptoms in pigs.

The noxious gas from feces is produced by the unabsorbed nutrients fermented by intestinal microbiota (79). Decrease the fermentation substrate by improving nutrient digestibility and regulate the fermentation process by regulating intestinal microbiota are strategies to reduce fecal noxious gas emission (32, 80). In this study, we observed that feeding weaned pigs with TA containing diet improved apparent nutrient digestibility and upregulated the abundance of beneficial microbiota in feces. We speculated that the reduction of fecal NH_3_ emission can be explained by the improvement in nutrient digestibility and the increase in beneficial microbiota abundance.

The total protein, albumin, and globulin concentrations *in vivo* are always linked to nutritional status (81, 82). In this study, dietary supplementation of TA had no significant effects on serum total protein, albumin, and globulin concentrations. Similarly, several studies reported that feeding weaned pigs with tributyrin containing diet did not affect the concentrations of total protein, globulin, and albumin in serum (2, 39, 70, 83). The effect of anise on serum total protein, albumin, and globulin concentrations are not yet known, but at least, it does not generate any damaging effect.

Tributyrin supplementation has been reported to regulate blood lipids (39, 84). Butyrate could promote the secretion of the main proteins that make up HDL-C, thus promoting the synthesis of HDL-C (85, 86). Xiong et al. (87) noted that feeding lipopolysaccharide-challenged broiler chicks with tributyrin containing diet increased serum HDL-C concentrations. In addition, feeding weaned pigs with anise containing herbal mixture also has been reported to increase the serum HDL-C concentrations, whereas decrease the serum total cholesterol concentrations. However, in this study, weaned pigs fed the diet supplemented with TA had no effects on serum total cholesterol, triglyceride, and HDL-C concentrations, which was affirmed by the studies of Dell'Anno et al. (83) and Weber et al. (88). Therefore, as far as the results obtained in this study were concerned, the contribution of TA to the regulation of serum lipid metabolism parameters was limited.

## Conclusion

Our findings confirmed that TA supplementation would improve growth performance and reduce fecal ammonia emission through improving nutrient digestibility, which was attributed to the increase of jejunal villus height and the regulation of fecal microbiota. Dose of TA at 0.15% seems suitable to be used in the diet of weaned pigs.

## Data availability statement

The data presented in this study are deposited in the figshare repository, accession number https://doi.org/10.6084/m9.figshare.21626027.v3.

## Ethics statement

The protocol (DK-1-2034) of this study was approved by the Animal Care and Use Committee of Dankook University (Cheonan, South Korea).

## Author contributions

IK, KL, and JS: conceptualization and methodology. DD and SL: formal analysis. DD, HL, SM, and KH: writing—original draft preparation. DD and JS: investigation. IK: supervision. DD, HL, SM, KH, and IK: writing—reviewing and editing. All authors reviewed the manuscript.
